# Phosphorylation of MAD2 at Ser195 Promotes Spindle Checkpoint Defects and Sensitizes Cancer Cells to Radiotherapy in ATM Deficient Cells

**DOI:** 10.3389/fcell.2022.817831

**Published:** 2022-03-02

**Authors:** Yang Wang, Tianyu Yu, Yi Han, Yazhi He, Yiran Song, Leiming Guo, Liwei An, Chunying Yang, Feng Wang

**Affiliations:** ^1^ Department of Gastroenterology, Shanghai 10th People’s Hospital, Tongji University School of Medicine, Shanghai, China; ^2^ Department of General Surgery, Pudong New Area Gongli Hospital Affiliated to Naval Military Medical University, Naval Military Medical University, Shanghai, China; ^3^ Department of R&D, Shanghai Creative Immune Therapeutics Co., Ltd, Shanghai, China; ^4^ Central Laboratory, Shanghai Putuo District People’s Hospital, Tongji University School of Medicine, Shanghai, China

**Keywords:** ATM kinase, mad2, phosphorylation, checkpoint defect, DNA damage repair

## Abstract

The spindle assembly checkpoint (SAC) is a critical monitoring device in mitosis for the maintenance of genomic stability. Specifically, the SAC complex comprises several factors, including Mad1, Mad2, and Bub1. Ataxia-telangiectasia mutated (ATM) kinase, the crucial regulator in DNA damage response (DDR), also plays a critical role in mitosis by regulating Mad1 dimerization and SAC. Here, we further demonstrated that ATM negatively regulates the phosphorylation of Mad2, another critical component of the SAC, which is also involved in DDR. Mechanistically, we found that phosphorylation of Mad2 is aberrantly increased in ATM-deficient cells. Point-mutation analysis further revealed that Serine 195 mainly mediated Mad2 phosphorylation upon ATM ablation. Functionally, the phosphorylation of Mad2 causes decreased DNA damage repair capacity and is related to the resistance to cancer cell radiotherapy. Altogether, this study unveils the key regulatory role of Mad2 phosphorylation in checkpoint defects and DNA damage repair in ATM-deficient cells.

## Introduction

Chromosome complementarity is naturally present in eukaryotic cells. During cell division, the gain or loss of chromosomes leads to abnormal chromosome numbers, which is termed aneuploidy and has been documented as one of the predispositions of tumorigenesis (Ganem et al., 2007; Storchova and Kuffer, 2008; Torres et al., 2010; Tang et al., 2011). To avoid abnormal chromosomes occurring, cells have evolved several checkpoints, including the DNA damage checkpoint (DDC), the DNA replication checkpoint (DRC), and the spindle assembly checkpoint (SAC), to ensure the genomic integrity of cells. SAC, also known as a mitotic checkpoint, plays a crucial role in ensuring the correct separation of chromosomes and the stability of genetic information. The SAC prevents the anaphase-promoting complex/cyclosome (APC/C) ubiquitin ligase from recognizing and securing cyclin B, ensuring chromosomes are properly attached to spindle microtubules (Bharadwaj and Yu, 2004; Musacchio and Salmon, 2007; Santaguida and Musacchio, 2009). In the prometaphase of the cell cycle, kinetochore, without microtubule attachments, recruits evolutionarily conserved proteins such as Aurora-B, Bub1, Bub3, BubR1/Mad3, Mad1, Mad2, Cad20, and MPS1, thus activating SAC to prevent the cells from entering anaphase (Li and Nicklas, 1995; Mora-Santos et al., 2016; Overlack et al., 2017; Raaijmakers et al., 2018). Among them, Mad2, Cdc20, Mad3 (also named BubR1 in some species), and Bub3 form the mitotic checkpoint complex (MCC), which are mainly responsible for inhibiting APC/C, leading to cell cycle arrest (Yu, 2006; Musacchio and Salmon, 2007; Chao et al., 2012). Accordingly, dysregulation of these proteins, either upregulation or downregulation, results in the breakdown of SAC and eventually genomic instability (Schuyler et al., 2012).

The mitotic arrest deficiency 2 (Mad2) is a SAC protein with two natural folding states, namely open conformer (O-Mad2) and close conformer (C-Mad2) (Luo et al., 2004). The transition of O-Mad2 to C-Mad2 is a key determinant for the assembly of a core complex required for activation of SAC between Mad2 and Mad1 (Yu, 2006; Yang et al., 2008). Besides, the Mad2 transition plays an important role in the subsequent inhibition of APC/C by binding to Cad20 (Luo et al., 2002) and is suspected as a mediator determining the metaphase–anaphase transition in mitosis (Varetti et al., 2011). In eukaryotic cells, another intrinsic mechanism maintaining genome stability is DNA damage response (DDR) (Kastan, 2008). As one of the key regulators in DDR, ataxia-telangiectasia mutated (ATM) recognizes DNA damage sites and phosphorylates histone H2AX, followed by recruiting the RAD50/MRE11/NBS1 complex to the breakpoints, thus initiating the DNA damage signaling cascade and repair process (Guleria and Chandna, 2016). In addition, ATM has also been reported to regulate diverse processes *via* phosphorylating distinct substrates, including checkpoints kinases 1,2 (CHK1,2) and p53 (Squatrito et al., 2010; Serrano et al., 2013; Zhang et al., 2014).

Despite intensive studies focusing on the importance of ATM in response to DDR, growing evidence has suggested the ATM's new role in mitosis. In this regard, we previously have proven that ATM kinase is activated in mitosis in the absence of DNA damage by Aurora-B-mediated Serine 1403 phosphorylation and also participated in SAC activation partially by regulation of Bub1 activity (Yang et al., 2011; Yang et al., 2012). In addition, it has been reported that changes in Mad2 levels not only affected the function of SAC, leading to increased chromosome loss and mitotic arrest (Rossio et al., 2010; Barnhart et al., 2011), but also promoted aneuploidy and induced tumorigenesis (Sotillo et al., 2010; Schvartzman et al., 2011). Nevertheless, whether ATM and Mad2 have a regulatory interrelationship during the cell cycle remains elusive. Outstanding work has demonstrated that *in vitro* artificially produced phosphorylated Mad2 leads to its protein architectures, which in turn affects its activity *in vivo* (Kim et al., 2010). However, the regulatory mechanisms that modulate Mad2 remain unknown. In this study, we found that human Mad2 is a phosphorylatable protein naturally occurring in various cells, and its phosphorylation level is negatively regulated by ATM.

## Materials and Methods

### Cell Lines and Culture

HeLa, 293FT, MCF7, and Panc-1 cells (American Type Culture Collection, Manassas, VA) were cultured in Dulbecco's modified Eagle's medium supplemented with 10% fetal bovine serum, 4 mM of l-glutamine, and 50 μg/ml of penicillin/streptomycin (all from Gibco, Carlsbad, CA). The simian virus 40-transformed human fibroblast cell lines GM9607 and GM0637 cells (National Institute of General Medical Sciences Human Mutant Cell Repository, Camden, NJ) were cultured in Roswell Park Memorial Institute 1640 medium supplemented with 10% fetal bovine serum and 50 μg/ml of penicillin/streptomycin. All cells were maintained in 5% CO_2_ at 37°C.

### Analysis of the Spindle-Assembly Checkpoint by Flow Cytometry

Approximately 10^6^ cells were trypsinized, washed, and resuspended in 70% ethanol at −20°C. Subsequently, cells were washed by phosphate-buffered saline with neither Ca^2+^ nor Mg^2+^ (D-PBS). After blocking in the D-PBS with 1% bovine serum albumin for 30 min, cells were incubated with Alexa Fluor® 488 Mouse monoclonal to Histone H3 (phospho S10) (1:100, ab197502, Abcam) at room temperature in the dark for recognition of mitotic cells. After 3 h, cells were washed three times with D-PBS by centrifugation, stained with 50-μg/ml propidium iodide (PI; C1052, Beyotime) for 30 min at 37 °C, and finally analyzed by FACSCanto II flow cytometer (BD Biosciences, San Jose, CA, USA). The percentage of mitotic cells was quantified by Flowjo software (Tree Star).

### Plasmids

The construction of Mad1-related plasmids was described in our previous study (Yang et al., 2014). Mad2 and related mutant plasmids were made using Gateway Technology. Briefly, the Mad2 complementary DNA was subcloned into pDONR221, pLVpuro-CMV-N-3Xflag (addgene#123223), and pDEST-CMV-N-EGFP (addgene#122842) vectors. DR-GFP (DR-U2OS), EJ5-GFP, and I-SceI plasmids were described in our previous study (An et al., 2018). All constructs were confirmed by DNA sequencing. The primers are shown in Table 1.

**TABLE 1:** List of primers used in this manuscript.

Primer’s name	Sequences
Flag-Mad2-WT-F	5′-GGG​GAC​AAG​TTT​GTA​CAA​AAA​AGC​AGG​CTT​AAT​GGC​GCT​G
	CAGCTCT-3′
Flag-Mad2-WT-R	5′-GGG​GAC​CAC​TTT​GTA​CAA​GAA​AGC​TGG​GTT​TCA​GTC​ATT​G
	ACA​GGA​ATT​TTG​TAG​GCC-3′
GFP-Mad2-WT-F	5′-GGG​GAC​AAG​TTT​GTA​CAA​AAA​AGC​AGG​CTT​AAT​GGC​GCT​G
	CAGCTCT-3′
GFP-Mad2-WT-R	5′-GGG​GAC​CAC​TTT​GTA​CAA​GAA​AGC​TGG​GTT​TCA​GTC​ATT​G
	ACA​GGA​ATT​TTG​TAG​GCC-3′
Flag-Mad2-S195A-F	5′-CAA​AGT​AAA​TGC​CAT​GGT​GGC​CTA​CAA​AAT​TCC-3′
Flag-Mad2-S195A-R	5′-AGG​CCA​CCA​TGG​CAT​TTT​ACT​TTG​TGG​ATT​GTA​G-3′
Flag-Mad2-S195D-F	5′-CAA​AGT​AAA​TGC​CAT​GGT​GGC​CTA​CAA​AAT​TCC-3′
Flag-Mad2-S195D-R	5′-AGG​CCA​CCA​TGG​CAT​TTA​CTT​TGT​GGA​TTG​TAG-3′
GFP-Mad2-S195A-F	5′-CAA​AGT​AAA​TGC​CAT​GGT​GGC​CTA​CAA​AAT​TCC-3′
GFP-Mad2-S195A-R	5′-AGG​CCA​CCA​TGG​CAT​TTT​ACT​TTG​TGG​ATT​GTA​G-3′
GFP-Mad2-S195D-F	5′-CAA​AGT​AAA​TGC​CAT​GGT​GGC​CTA​CAA​AAT​TCC-3′
GFP-Mad2-S195D-R	5′-AGG​CCA​CCA​TGG​CAT​TTA​CTT​TGT​GGA​TTG​TAG-3′
GFP-Mad2-S120A-F	5′-CAG​AGA​AAA​GGC​ACA​GAA​AGC​TAT​CCA​GGA​TGA​AAT​C-3′
GFP-Mad2-S120A-R	5′-TAG​CTT​TCT​GTG​CCT​TTT​CTC​TGG​GTG​CAC​TGT​C-3′
GFP-Mad2-S170A-F	5′-ATG​GGA​AGA​GGC​CGG​ACC​ACA​GTT​TAT​TAC​CAA​TTC-3′
GFP-Mad2-S170A-R	5′-ACT​GTG​GTC​CGG​CCT​CTT​CCC​ATT​TTT​CAG​GTA​C-3′
GFP-Mad2-S178A-F	5′-TAT​TAC​CAA​TGC​CGA​GGA​AGT​CCG​CCT​TCG​TTC-3′
GFP-Mad2-S178A-R	5′-GGA​CTT​CCT​CGG​CAT​TGG​TAA​TAA​ACT​GTG​GTC-3′
GFP-Mad2-S185A-F	5′-CCG​CCT​TCG​TGC​CTT​TAC​TAC​TAC​AAT​CCA​CAA​AAG-3′
GFP-Mad2-S185A-R	5′-TAG​TAG​TAA​AAG​GCA​CGA​AGG​CGG​ACT​TCC​TCA​G-3′
HA-Mad1-WT-F	5′-ACT​GGA​TCC​ACG​ATG​TAC​CCA​TAC​GAT​GTT​CCA​GAT​TAC
	GCT​ATG​GAA​GAC​CTG​GGG​GAA​AAC​ACC​A-3′
HA-Mad1-WT-R	5′-AGC​TCT​AGA​CTA​CGC​CAC​GGT​CTG​GCG​GCT​GAA​GAG-3′
HA-Mad1-S214A-F	5′-GAA​CTC​CAG​GCC​GCA​CAA​GAA​GCA​AGA​GCA​GAC​CAC​G
	AGCAGC-3′
HA-Mad1-S214A-R	5′-GCT​GCT​CGT​GGT​CTG​CTC​TTG​CTT​CTT​GTG​CGG​CCT​G
	GAGTTC-3′
HA-Mad1-S214E-F	5′-GAA​CTC​CAG​GCC​GAG​CAA​GAA​GCA​AGA​GCA​GAC-3′
HA-Mad1-S214E-R	5′-GCT​ACT​CGT​GGT​CTG​CTC​TTG​CTT​CTT​GCT​CGG​CCT
	GGAGTTC-3′

### Reagents and Antibodies

Nocodazole and Ku55933 were ordered from Selleckchem (Texas, USA). Lambda Protein phosphatase (λPPase) was bought from Sigma-Aldrich Corporation. The anti-ATM (1:1,000, ab201022, Abcam), anti-phospho-ATM (1:1,000, ab81292, Abcam), anti-Mad2 (1:1,000, ab70385, Abcam), anti-Mad1 (1:1,000, ab201022, Abcam), and anti-Cdc20 antibodies (1:1,000, ab183479, Abcam) were bought from Abcam (Cambridge, MA). The anti-HA (1:1,000, 3724T, Cell Signaling Technology), anti-Mad2 (1:1,000, 4636S, Cell Signaling Technology), and the HRP-conjugated secondary antibodies were purchased from Cell Signaling Technology (Danvers, MA). The anti-Flag antibody (1:1,000, F3165, Sigma) was purchased from Sigma. The anti-Mad2 antibody (1:1,000, YT2618, Immunoway) was bought from Immunoway. The anti-GFP (1:1,000, sc-9996, Santa Cruz), anti-Mad2 (1:1,000, sc-47747, Santa Cruz), anti-*p*-Thr (1:1,000, sc-5267, Santa Cruz), and anti-*p*-Ser antibodies (1:1,000, sc-81514, Santa Cruz) were bought from Santa Cruz Biotechnology. Goat anti-mouse lgG-H HRP (1:1,000, M21004L, Abmart) and goat anti-mouse lgG-L HRP (1:1,000, M21005S, Abmart) were purchased from Abmart.

### Retroviral ATM and Control Short Hairpin RNA Production

Vectors carrying the human ATM short hairpin RNA (shRNA) with the target sequence of 5′-AAG​CGC​CTG​ATT​CGA​GAT​CCT-3′ or nontargeting control with the sequence of 5′-TTC​TCC​GAA​CGT​GTC​ACG​T-3′ were purchased from Genechem (Shanghai, China). The recombinant viruses of human ATM shRNA and control shRNA were transiently transfected into 293T cells with GV115 viral vector packaging system (Genechem) consisting of pHelper1.0, pHelper2.0, and GV115. After 72 h, the supernatant filled with target viruses was harvested for titer analysis and subsequently transfected into HeLa cells. Polymerase chain reaction and Western blot analysis confirmed the knockdown level.

### Small Interfering RNA Transfection

Cells were transfected with ATM-specific small interfering RNA (siRNA) oligos (si-ATM: 5′-GCU​AUU​UAC​GGA​GCU​GAU​UTT-3′) (GenePharma, China) at 50 nmol/L final concentration using INTERFERin (Polyplus-transfection, France) as the transfection reagent. A scrambled siRNA (siScr: 5′-UUC​UCC​GAA​CGU​GUC​ACG​UTT-3′) (GenePharma, China) was applied as a negative control. Cells were harvested 72 h, followed by gene expression analysis by Western blot analysis.

### Western Blotting

Cells were scraped in PBS, pelleted, and lysed in NETN buffer (20-mM Tris-HCl, pH 8.0, 100-mM NaCl, 0.5% Nonidet P-40, and 1-mM ethylenediaminetetraacetic acid) supplemented with BitNuclease (Biotool) on ice for 15 min. Cell lysates were boiled after the addition of Lamelli buffer. Proteins were separated by sodium dodecyl sulfate–polyacrylamide gel electrophoresis gel and then transferred to the nitrocellulose membrane. After incubation with indicated primary and secondary antibodies, blots were filmed and detected using the Pierce chemiluminescence detection system.

### Immunoprecipitation

Our previous study described immunoprecipitation (An et al., 2020). Whole-cell lysates were incubated with Flag Agarose beads or protein A/G plus agarose beads conjugated with indicated antibodies overnight at 4°C, washed three times with NETN buffer. To avoid the noise of light or heavy chains, the immunoprecipitation assays, including goat anti-mouse immunoglobulin G heavy chain and goat anti-mouse immunoglobulin G light chain, were used as the secondary antibodies.

### Colony Formation Assay

HeLa cells transfected with empty vector, wild-type (WT), S195A, or S195D mutant form of Mad2 (1,000 cells per well) were plated to six-well plates, irradiated with the indicated doses of X-rays, and then further cultured for 12 days. After incubation with crystal violet for 60 min at room temperature, the colonies were imaged under a stereomicroscope and counted with ImageJ.

### DSB Reporter Assay

HeLa cells stably expressing the WT, S195A, or S195D mutant form of Mad2 were electroporated with the I-SceI expression construct (pCBASce) together with DR-GFP or EJ5-GFP reporter plasmid at 150 V, 975 μF using NEPA21 Super Electroporator (NEPA GENE). Cells were further recovered for 48 h after electroporation followed by flow cytometric analysis on a BD FACS CantoII Analyzer.

### Statistics

Unless otherwise stated, data for the statistical analysis are obtained from at least three independent experiments. The unpaired Student's t-test was used to evaluate statistical significance. Values of *p* ≤ 0.05 were considered statistically significant.

## Results

### ATM Deficiency Causes Severe SAC Defects in the Absence of DNA Damage

**FIGURE 1:**
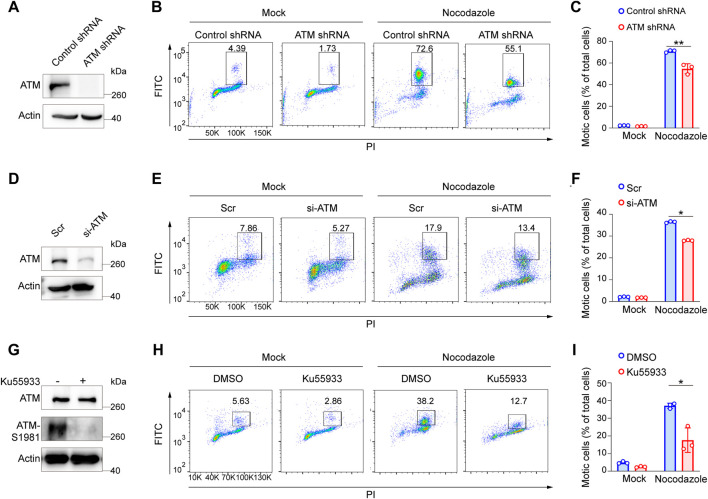
Elimination of ATM resulted in a severe defect in SAC in absence of DNA damage. **(A)** Immunoblotting analysis of ATM level in ATM shRNA and control shRNA cells. **(B)** FACS analysis of ATM shRNA and control shRNA cells treated with nocodazole for 16 h followed by flow cytometric using anti-phospho-H3. **(C)** Mean mitotic percentages of at least triplicate samples are shown. Error bars represent variations around averages. **(D)** Immunoblotting analysis of knockdown efficiency of ATM in Panc-1 cells transfected with ATM-specific siRNA for 48 h, scrambled siRNA as a positive control. **(E)** FACS analysis of Panc-1 cells transfected with scrambled siRNA or ATM-specific siRNA treated with or without nocodazole. **(F)** Mean mitotic percentages of at least triplicate samples are shown, and error bars represent variations around averages. **(G)** Immunoblotting analysis of ATM activity in Hela cells treated with KU-55933 for 2 h. **(H)** FACS analysis of HeLa cells treated with dimethyl sulfoxide or KU-55933 and nocodazole. **(I)** Mean mitotic percentages of at least triplicate samples described in **(B)**. Error bars represent variations around averages.

ATM deficiency has been associated with chromosomal instability, thus increasing the radiosensitivity (Shiloh, 2003). To further investigate the effects of ATM for the SAC process in the absence of DNA damage, we first infected HeLa cells with lentivirus harboring control shRNA or ATM shRNA to generate stable ATM knockdown cell lines (Figure 1A). Then, we treated cells with nocodazole to arrect cells in the mitotic phase. Interestingly, we reproducibly observed a significant decrease in the percentage of mitotic cells in ATM-deficient cells as compared with control WT cells, revealing a severely impaired activation of SAC (Figures 1B,C). To confirm this discovery, we repeated this assay in Panc-1 cell lines *via* siRNA-mediated knockdown approach (Figure 1D) and HeLa cells *via* ATM inhibitor. Consistently, we found that depletion of ATM resulted in dramatically reduced mitotic cell population (Figures 1E,F,H,I). Thus, these phenomena indicated that ATMs play a crucial role in maintaining the activation of SAC.

### ATM Deficiency Augments the Phosphorylation of Mad2 by Mediating Mad1 Serine 214 Phosphorylation

**FIGURE 2:**
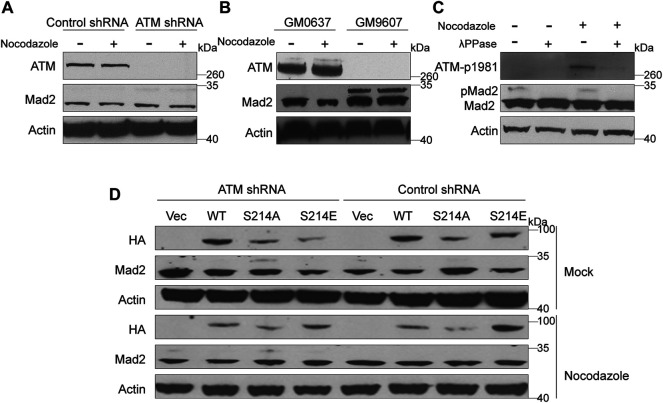
Elimination of ATM increased phosphorylation of Mad2. **(A)** Immunoblotting analysis of control or ATM shRNA cells in absence or presence of nocodazole. **(B)** Immunoblotting analysis in simian virus 40-transformed fibroblast cell lines GM9607 and GM0637. **(C)** Immunoblotting analysis of cells treated with mock or nocodazole in presence or absence of λPPase. **(D)** Immunoblotting analysis of HeLa cells transfected with vector, WT, S214A, or S214D mutant form of Mad1 of in presence or absence of nocodazole.

Mad2 is a mitotic factor downstream of the ATM during DDR, and its downregulation often impairs the function of SAC, causing shortened mitosis time (Kim et al., 2010; Yang et al., 2014). We assessed whether depletion of ATM would impact the function of Mad2. To this end, we first compared the expression level of Mad2 in GM9607 cells, a naturally ATM-deficient cell line (Yang et al., 2014), and GM0637. However, we did not observe any change in the total Mad2 protein level (Figure 2B). Interestingly, we found a clear Mad2 shift band that highly resembled post-translational modification (PTM) (Figure 2B). Authentically, this Mad2 shift band repeatedly appeared in ATM knockdown HeLa cells (Figure 2A), and Mad2 shift bands are inversely correlated with the ATM protein level, indicating that ATM deficiency may induce Mad2 PTM. It is worth mentioning that the emergence of the Mad2 shift band was not affected by nocodazole treatment.

ATM is a kinase that functions by regulating the phosphorylation of downstream proteins. Thus, we suspect that the shift band might be a manifestation of mad2 phosphorylation. To confirm our hypothesis, we used lambda protein phosphatase (λPPase) as a phosphorylation inhibitor on the total proteins of GM9607 cells. In addition, we also included GM0637, an ATM proficient cell line as a positive control, and examined the phosphorylation status of ATM-S1981, which is a well-known ATM protein serine site that can be autophosphorylated. Surprisingly, we found that both ATM-S1981 phosphorylation band and Mad2 shift band disappeared with λPPase treatment (Figure 2C), demonstrating that the shift band in Figures 2A,B (measured by antibody against Mad2) is phosphorylated Mad2.

Mad1 and Mad2 are two key SAC proteins. Our previous studies have proved that ATM-mediated Serine 214 phosphorylation of Mad1 promotes Mad1 homodimerization and heterodimerization with Mad2, which contributes to the activation of the SAC (Yang et al., 2014). To further investigate whether this process has an effect on Mad2 phosphorylation, we constructed three Mad1 plasmids, including HA-tagged WT, S214A (the seine to alanine mutant), which cannot be phosphorylated, and S214E (the seine to glutamic acid mutant), which is a mimic phosphorylated Mad1 (Figure 2D). These plasmids were transiently transferred into HeLa cells stably expressing ATM shRNA or control shRNA. We found that Mad2 phosphorylation was significantly higher in the ATM-deficient HeLa cells expressing S214A than that in other cells, indicating that Mad2 phosphorylation was negatively regulated by ATM.

### Mad2 Is Mainly Phosphorylated at Ser195 Upon ATM Depletion

**FIGURE 3:**
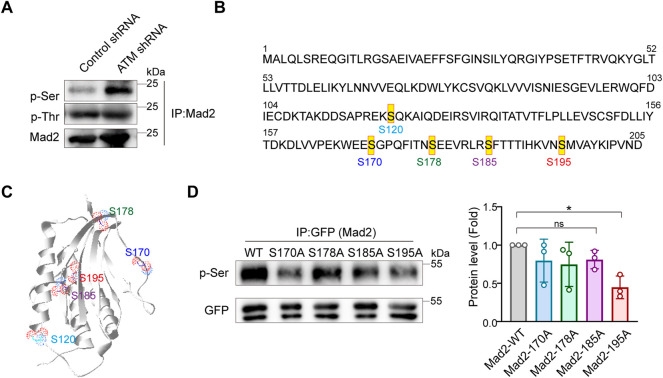
Mad2 is mainly phosphorylated at Ser195 in ATM-loss cells. **(A)** Immunoprecipitation assay of detecting abundance of Mad2 phosphorylation in control or ATM shRNA cells. **(B)** Some phosphorylated sites in Mad2. **(C)** Structures of Mad2 S120, S170, S178, S185, and S195 are shown as ball-and-stick. **(D)** Immunoprecipitation assay of HeLa cells transfected with GFP-tagged WT, S170A, S178A, S185A, or S195A mutants of Mad2. Exogenous proteins were immunoprecipitated with anti-GFP antibody followed by immunoblotting using indicated antibodies. Statistic analyses were done by *t*-test, and *p* values are presented.

Phosphorylation events mainly occur at serine or tyrosine residues of proteins. Next, to identify the key residues responsible for Mad2 phosphorylation upon ATM deletion, we examined the total serine or tyrosine phosphorylation on Mad2 using individual pan phospho-Tyr or phospho-Ser antibodies. Intriguingly, we observed dramatic upregulation of serine but not tyrosine phosphorylation in ATM knockdown cells, indicating that the Mad2 shift band in this study is mainly serine-phosphorylated Mad2 (Figure 3A). Therefore, next, we focused on several key serine residues in Mad2, including S170, S178, S185, and S195, which have been documented to be functioning in SAC complex formation (Kim et al., 2010) (Figures 3B,C). To further narrow down the phosphorylated site(s), we applied the point mutation strategy and generated Aline mutant on those residues, respectively. 293FT cells were transfected with plasmids encoding GFP-tagged Mad2^WT^, Mad2^S170A^, Mad2^S178A^, Mad2^S185A^, or Mad2^S195A^. All the GFP-tagged Mad proteins are transiently expressed. We performed immunoprecipitation using GFP antibody followed by Western blot using phospho-Ser antibodies. The results showed that S195A mutation greatly attenuated Mad2 phosphorylation (Figure 3D). Overall, these results demonstrated that Mad S195 is mainly phosphorylated upon ATM depletion.

### S195 Phosphorylation of Mad2 Regulates the Spindle Checkpoint

**FIGURE 4:**
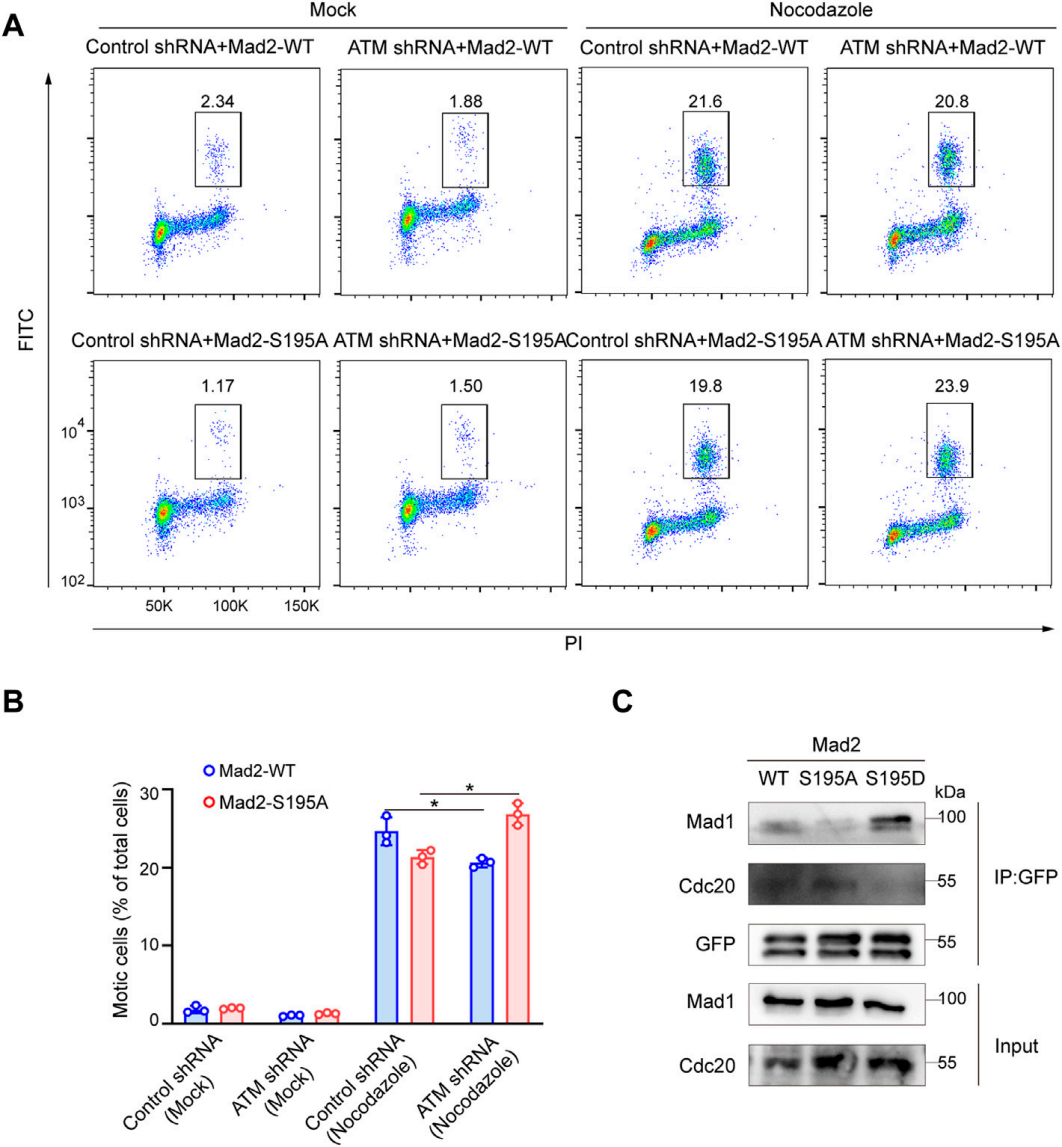
Phosphorylation of Mad2 Serine 195 regulated Spindle Checkpoint. **(A)** FACS analysis of HeLa cells transfected with vector, WT, S195A, or S195D mutant form of Mad2 and treated with nocodazole followed by flow cytometric anti-phospho-H3 staining. **(B)** Mean mitotic percentages (at least triplicate samples) and error bars represent variations around averages. **(C)** Co-immunoprecipitation assay of Mad2 complex in 293FT cells transfected with GFP-tagged S195A or S195D mutants of Mad2.

We next evaluated whether S195 phosphorylation of Mad2 is essential for the SAC process in ATM-deficient cells. To this end, we utilized flow cytometry to analyze the mitotic cell population in Hela cells ectopically expressing Mad2^WT^ or Mad2^S195A^. We found that S195A overexpression led to a much less mitotic cell population than Mad2^WT^ overexpression (Figures 4A,B). On the contrary, in ATM-deficient cells, overexpression of Mad2^S195A^ induced increased mitotic index than Mad2^WT^ cells after nocodazole treatment (Figures 4A,B). In addition to the S195A mutant, we also generated a phosphomimic mutant S195D to characterize assembly of CDC20-Mad1-Mad2 complex *via* co-immunoprecipitation assay. As shown in Figure 4C, Mad2^WT^ or Mad2^S195A^ can still form the complex with CDC20-Mad1. However, Flag-Mad2^S195D^ failed to bind to Cdc20 but with increased binding ability to Mad1 (Figure 4C). Taken together, these data suggested that S195 phosphorylation of Mad2 plays a critical role in SAC after ATM loss.

### pMad2^S195^ Impairs DNA Repair Capacity and Confers Cancer Cell Sensitivity to Radiotherapy

**FIGURE 5:**
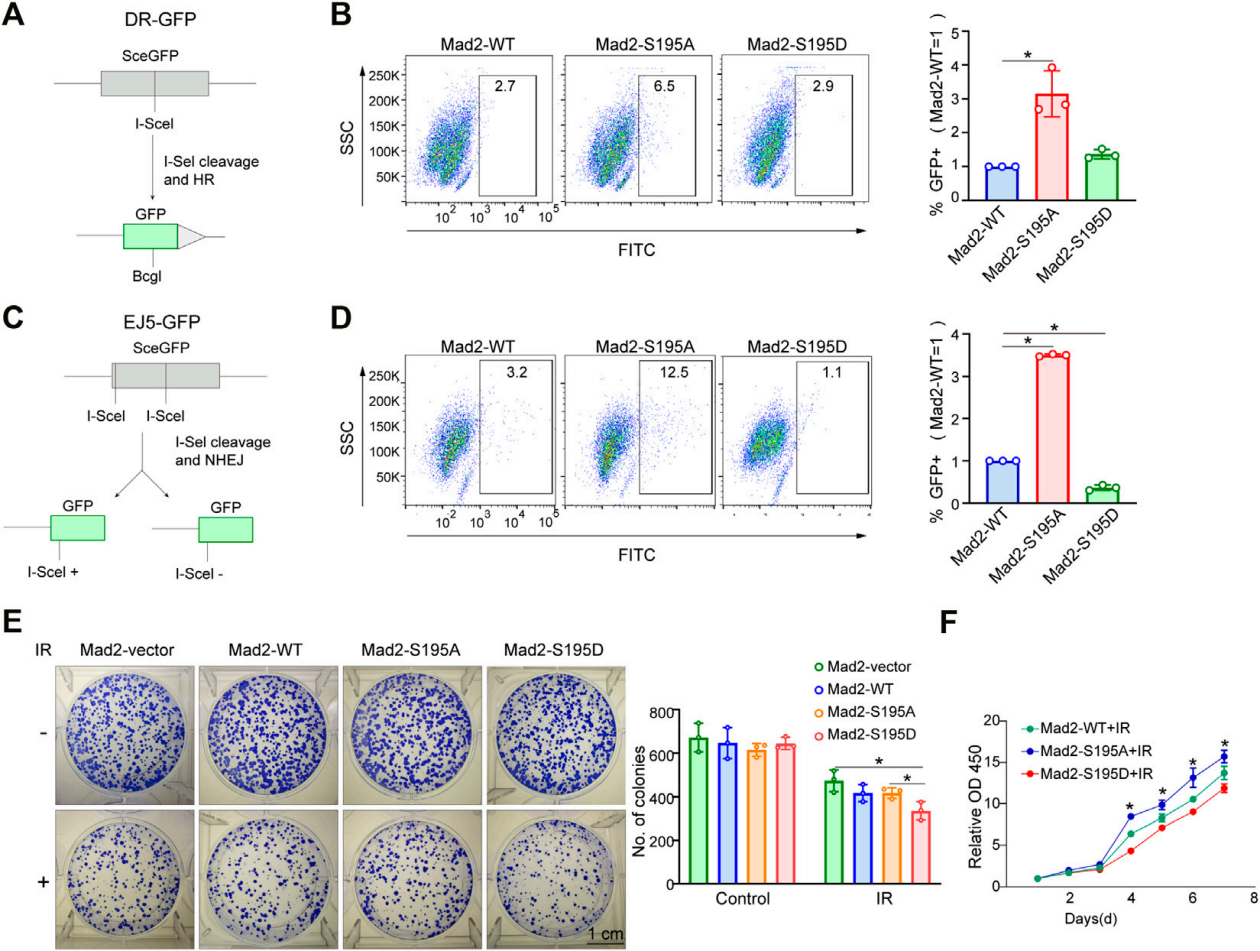
Mad2 phosphorylation impaired DSB repair. **(A,B)** Homology-directed repair assay in control shRNA or ATM shRNA cells subjected to vector, WT, S195A, or S195D transfection. **(C,D)** Nonhomologous end-joining assay in control shRNA or ATM shRNA cells subjected to vector, WT, S195A, or S195D transfection. **(E)** Colony formation assay of control shRNA or ATM shRNA cells transfected with empty vector only, WT, S195A, or S195D with or without interventional radiology treatment. Mean ± SD, Student's t-test, n = 3, *p < 0.05. **(F)** CCK-8 assay analysis of cell proliferation abilities in control shRNA or ATM shRNA cells subjected to vector, WT, S195A, or S195D transfection.

The cell cycle phase determines a cell's relative radiosensitivity. Cells in the G (2)-M phase are more radiosensitive compared with those in other phases (Pawlik and Keyomarsi, 2004). Mad2 phosphorylation decreased the proportion of cells in the M phase. To systemically evaluate the effect of phosphorylation of Mad2 on DNA repair and cancer cell sensitivity to radiotherapy, we first utilized a well-established DNA double-strand break (DSB) repair reporter assay to assess the ability of each mutant in promoting DNA repair (Figures 5A,C). We found that Mad2 phosphorylation (S195D) significantly inhibited DNA damage repair *via* both homologous recombination (HR) and nonhomologous end-joining (Figures 5B,D). In contrast, S195A mutant greatly stimulated the DSB repair process. Consistent with this observation, further colony formation assay revealed that expression of S195A mutant led to more colony numbers than the expression of WT and S195D, indicating that cells with Mad2-S195D increase cancer cell sensitivity to radiotherapy (Figures 5E,F).

## Discussion

Mad2 possesses typical bimodal protein with two natural folded structures, O-Mad2 and C-Mad2. Compared with the O-Mad2 architecture, the C-Mad2 architecture is more stable and has a stronger affinity with Cdc20, which inhibits the activation of APC/C. Therefore, C-Mad2 has been considered to be an activated form of Mad2. A few years ago, a splendid work showed that the activity of Mad2 can be regulated by exogenous Serine 195 phosphorylation in its C-terminal region (Kim et al., 2010). Although the phospho-mimicking Mad2^S195D^ mutant is easier to bind to high-affinity ligands such as Mad1 and MBP1, it inhibits the spontaneous formation of C-Mad2 while failing to bind to the Cdc20, a relatively low-affinity ligand. In addition, the existence of Mad2^S195D^ significantly caused severe damages to SAC complexes (Kim et al., 2010). Thus, the underlying mechanism of Mad2^S195D^, inhibiting Mad2 function and destroying the SAC, is to differentially alter its ability of binding to Mad1 or Cdc20 by adjusting its protein structure. However, this study did not specifically investigate the existence of endogenous phosphorylation of Mad2 and is according to certain pathways.

ATM is a canonical DNA damage checkpoint protein. We recently found that ATM is also essential for maintaining genome stability in mitosis (Yang et al., 2012; Yang et al., 2014). In addition, our previous research has proved that ATM plays a critical role in SAC by phosphorylating Bub1 on Ser314, thus activating the SAC (Yang et al., 2011). Mad2 is another member of the kinetochore protein complex aside from Bub1. More interestingly, we also found that ATM affects Mad1 and Mad2 complex formation by phosphorylating Mad1 on Serine 214 (Yang et al., 2014). Therefore, we hypothesized that Mad2 may be the direct substrate of ATM kinase, as it always modulates the same pathway by phosphorylating a serious protein. In this study, although there are no phosphate-specific antibodies against Mad2 S195, we found that endogenous Mad2 phosphorylated forms highly regulated by ATM protein level are readily detected by conventional antibodies.

**FIGURE 6:**
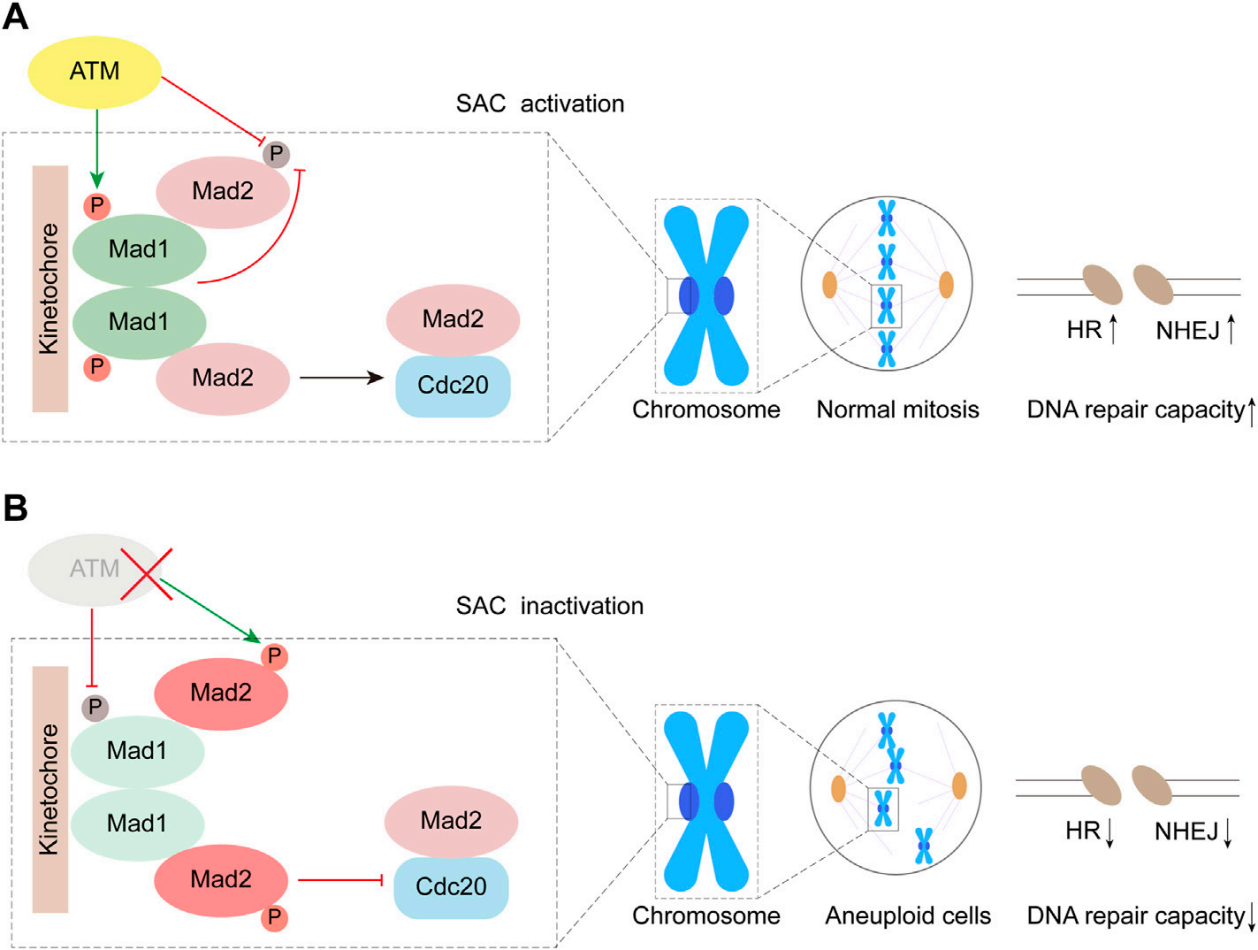
Working model. ATM inhibits phosphorylation of Mad2 by mediating Mad1 Serine 214 phosphorylation.

Mad1 is one of the evolutionarily conserved core proteins for SAC, which utilizes its Mad2 interaction motif (MIN) located at the middle region to form a complex with Mad2. During mitosis, the components of the SAC complexes are recruited to unattached kinetochores, and then, the molecular conformation of Mad2 dwelling in the complex changes from a dormant O-Mad2 to a functional C-Mad2, which is one of the key signal amplifying mechanisms for the activation of the SAC. In our previous study, we reported that the heterodimerization consisting of Mad1 and Mad2 was highly regulated by the S214 phosphorylation (S214p) site directly mediated by ATM, and the maintenance of Mad1-S214p is of great significance for preserving SAC function (Yang et al., 2014). In this study, our data indicated that the phosphorylation of Mad2 was observably promoted by S214A in the absence of ATM, whereas the plasmid of S214E and WT significantly inhibited Mad2 phosphorylation, which should occur in ATM-deficient cells. However, despite the presence of S214A, Mad2 no longer exhibited significant levels of phosphorylation in the presence of ATM (Figure 2D). Therefore, based on this phenotype, there may be some different pathways regulating Mad2 phosphorylation. In cells with deficient ATM, the amount of non-phosphorylated Mad2 may be directly determined by the level of S214p in Mad1, indicating that Mad1 with S214p is indispensable for ensuring Mad2 activity. When ATM is proficient in cells, ATM may inhibit Mad2 phosphorylation by urging the amount of S214p in Mad1 (Figure 6). Besides, it is also possible that ATM might directly act on Mad2 or regulate some involved kinases through the non-Mad1 pathway to maintain the non-phosphorylation of Mad2 (Kastan and Lim, 2000; Chen et al., 2021; Jiang et al., 2022).

Mad2 also plays a critical role in the cellular response to DNA damage (Dotiwala et al., 2010). The Mad2 expression was negatively related to the expression of γH2AX. The N-terminal domain of the Mad2 protein is important in the response to DNA damage (Fung et al., 2008). The overexpression of Mad2 promotes chemosensitivity to anticancer drugs in some tumor cells (Fung et al., 2008; Nascimento et al., 2016). However, other studies reported that Mad2 depletion causes mitotic checkpoint defects to promote mitotic exit, conferring cancer cells sensitive to anticancer drugs (Nascimento et al., 2014; Nascimento et al., 2016). In this study, we found that the Mad2 C-terminal region also exerts a role in DNA damage repair (Figure 5). Mad2 phosphorylation decreases M phase cells but decreases DNA repair capacity. Thus, Mad2 phosphorylation causes tumor cells to be sensitive to radiotherapy.

In general, our data demonstrate that, in addition to *in vitro*, Mad2 is a protein with a phosphorylated form also *in vivo*, and its phosphorylation level is regulated by ATM. We also consider that the phosphorylation of Mad2 may play a prominent role in DNA repair pathways. In future studies, we will pursue the kinase involved in Mad2 phosphorylation.

## Data Availability

The original contributions presented in the study are included in the article/supplementary material; further inquiries can be directed to the corresponding authors.

## Abbreviations

APC/C, anaphase-promoting complex or cyclosome; ATM, Ataxia-Telangiectasia Mutated kinase; CHK1, checkpoints kinases 1; CHK2, checkpoints kinases 2; DDC, DNA damage checkpoint; DRC, DNA replication checkpoint; DDR, DNA damage response; MCC, mitotic checkpoint complex; Mad2, mitotic arrest deficiency; PTM, post-translational modification; SAC, spindle assembly checkpoint; WT, wild-type.
